# CHK2 kinase in the DNA damage response and beyond

**DOI:** 10.1093/jmcb/mju045

**Published:** 2014-11-17

**Authors:** Laura Zannini, Domenico Delia, Giacomo Buscemi

**Affiliations:** 1 Department of Experimental Oncology, Fondazione IRCCS Istituto Nazionale dei Tumori, Via Amadeo 42, 20133 Milan, Italy; 2 Department of Biosciences, University of Milan, via Celoria 26, 20133 Milan, Italy

**Keywords:** DNA damage, checkpoints, apoptosis, genomic stability

## Abstract

The serine/threonine kinase CHK2 is a key component of the DNA damage response. In human cells, following genotoxic stress, CHK2 is activated and phosphorylates >20 proteins to induce the appropriate cellular response, which, depending on the extent of damage, the cell type, and other factors, could be cell cycle checkpoint activation, induction of apoptosis or senescence, DNA repair, or tolerance of the damage. Recently, CHK2 has also been found to have cellular functions independent of the presence of nuclear DNA lesions. In particular, CHK2 participates in several molecular processes involved in DNA structure modification and cell cycle progression. In this review, we discuss the activity of CHK2 in response to DNA damage and in the maintenance of the biological functions in unstressed cells. These activities are also considered in relation to a possible role of CHK2 in tumorigenesis and, as a consequence, as a target of cancer therapy.

## Introduction

From 50000 to 500000: these are the estimates of the number of insults to the nuclear DNA of each cell of our body every day, as a result of normal metabolism. This number is further increased by the genotoxic effects of air pollution, cigarette smoking, food additives, toxins, solar ultraviolet radiation, X-ray exams, and nuclear plant disasters (Ciccia and Elledge, 2010). Indeed, free oxygen radicals that arise during metabolism or exposure to ionizing radiation (IR) can break the phosphodiester bonds in the backbone of the DNA helix. Similarly, alkylating agents and ultraviolet irradiation can distort the DNA helix and lead to chromosomal breakage. When the lesion damages only one of the two strands of the double helix, a single strand break occurs. When two of these breaks are close and on opposite strands, a double-strand break (DSB) can happen (Ciccia and Elledge, 2010). DSBs occur infrequently (∼10 per cell for each day), but are dangerous because they can lead to chromosomal rearrangements or loss of genetic material. For this reason and for the intensity of the cellular response to them, DSBs are widely studied.

**Figure 1:**
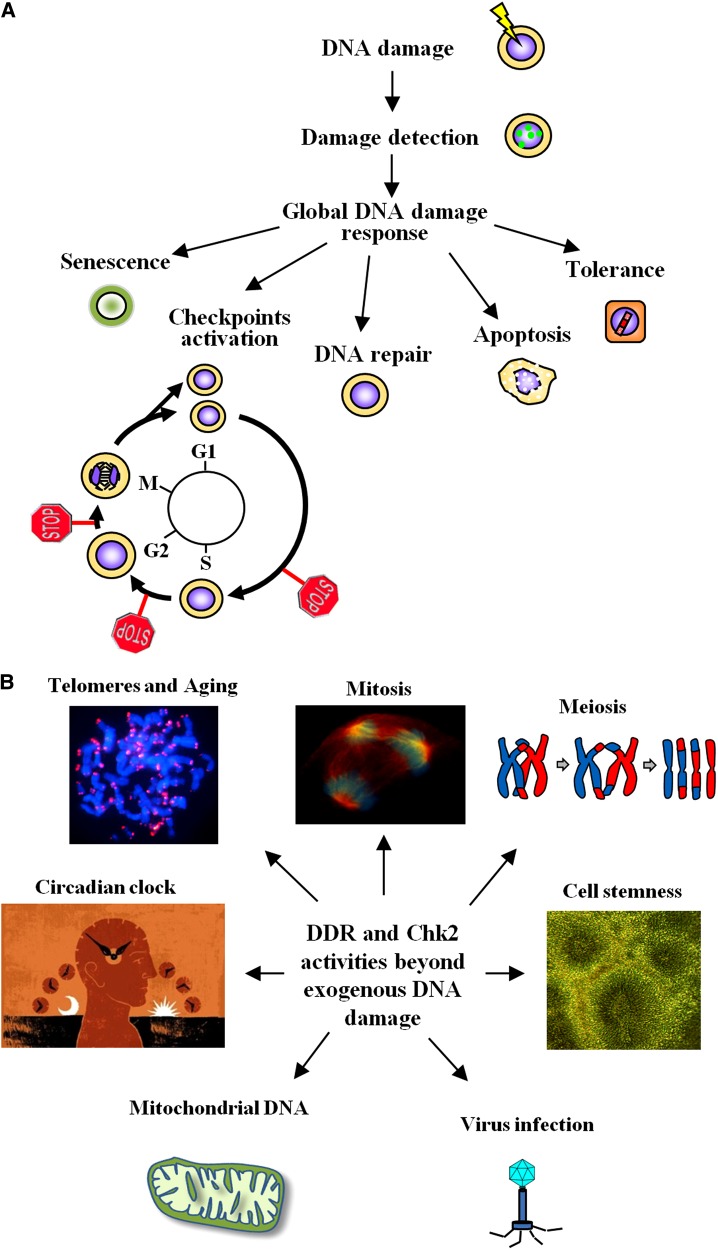
The DNA damage response and CHK2 functions in human cells. (**A**) When a lesion is detected, the DNA damage response promotes the appropriate cellular reaction that could be senescence, checkpoint activation, DNA repair, apoptosis, or tolerance of the damage. (**B**) Overview of common DDR and CHK2 activities related to DNA structure and cell cycle progression.

To combat alterations to the nuclear DNA, all organisms have developed a complex yet efficient system for repairing damage and eliminating cells that are beyond repair. Called the DNA damage response (DDR) (Ciccia and Elledge, 2010), this cascade of events involves, in eukaryotes, hundreds of proteins that sense lesions, arrest the cell cycle at checkpoints, repair DNA or, in presence of irreparable damage, initiate a program of permanent duplication arrest (senescence) or suicide (apoptosis) (Figure 1A). The DDR is also involved in other cellular functions independent of the presence of nuclear DNA damage. In particular, DDR proteins help to preserve DNA integrity by participating in telomere length maintenance (O'Sullivan and Karlseder, 2010), mitochondrial DNA repair (Alexeyev et al., 2013), and viral DNA processing (Turnell and Grand, 2012). The DDR is also involved in regulating the cell cycle, mitosis (Heijink et al., 2013) and meiosis (Richardson et al., 2004), and in the repair of DSBs at variable (V), diversity (D) and joining (J) gene segment (V(D)J) recombination and during class-switch recombination, two reactions necessary for antigen receptor assembly by lymphocytes (reviewed in Callen et al. (2007)). The DDR is also linked to the circadian clock: genotoxic agents alter circadian parameters and circadian proteins are involved in the response to genotoxic lesions (Sancar et al., 2010). Moreover, a robust response to DNA damage is critical for the stability of the stem cell genome, important for ensuring an accurate differentiation program (Nagaria et al., 2013).

The DDR (Figure 1A) is triggered when sensor proteins, which constantly scan the DNA, find structural distortions or breaks (Ciccia and Elledge, 2010). They mark these events and attract to these sites two enzymes, namely serine/threonine protein kinase ATM (also called ataxia telangiectasia mutated) and serine/threonine protein kinase ATR (also called ataxia telangiectasia and Rad3-related protein), which belong to the phosphatidylinositol-3 kinase (PI3K) family and are the apical (initiating) kinases of the DDR cascade. Whereas ATM seems to be activated primarily by DSBs (Shiloh and Ziv, 2013), ATR is mainly involved in the response to stalled replication forks (Marechal and Zou, 2013), although it can also participate in the DDR to DSBs. Upon DNA damage, ATM and ATR phosphorylate a multitude of substrates to induce the required cellular response (Ciccia and Elledge, 2010). Initially, to transduce the DNA damage signal, they cooperate with two other classes of proteins: the checkpoint mediators and the transducer kinases. Checkpoint mediators (MDC1, 53BP1, and BRCA1 for ATM (Shiloh and Ziv, 2013); and TopBP1 and claspin for ATR) contribute to the activation of ATM and ATR by indirectly binding to the lesions and facilitating recruitment of DDR factors to the damaged sites (Canman, 2003; Marechal and Zou, 2013). Checkpoint mediators accumulate at sites of DNA damage in foci, structures that spread up to 2 Mb around the lesion, and recruit proteins to facilitate break repair (Bekker-Jensen and Mailand, 2010).

The other class of proteins, the transducer kinases, is involved in spreading of the DNA damage signal through a phosphorylation cascade. Two transducer kinases are known: CHK2 for ATM (Matsuoka et al., 2000) and CHK1 for ATR (Kumagai et al., 2004). They phosphorylate effector proteins, which are the executors of DDR functions and may also be phosphorylated by ATM and ATR and by other kinases. In this way, the transducer kinases enhance or redirect the ATM-ATR response.

In the case of DSBs, the spreading activity is primarily played by the nuclear serine/threonine protein kinase Chk2 (CHK2). Here, we review CHK2 activation and activity in the cellular response to DNA damage and analyze emerging roles for this protein in other crucial cellular pathways that require the maintenance or restoration of genome integrity (Figure 1B). Indeed, similarly to other DDR proteins, CHK2 is involved in the control of mitosis and meiosis progression and in the maintenance of stem cell genomic stability. Moreover CHK2 has been found to interact with viral proteins during infections and to be involved in the response to mitochondrial DNA damage. In addition, it has been found that CHK2 regulates circadian proteins which in turn regulate CHK2 itself.

## CHK2 structure, activation, and inactivation

**Figure 2:**
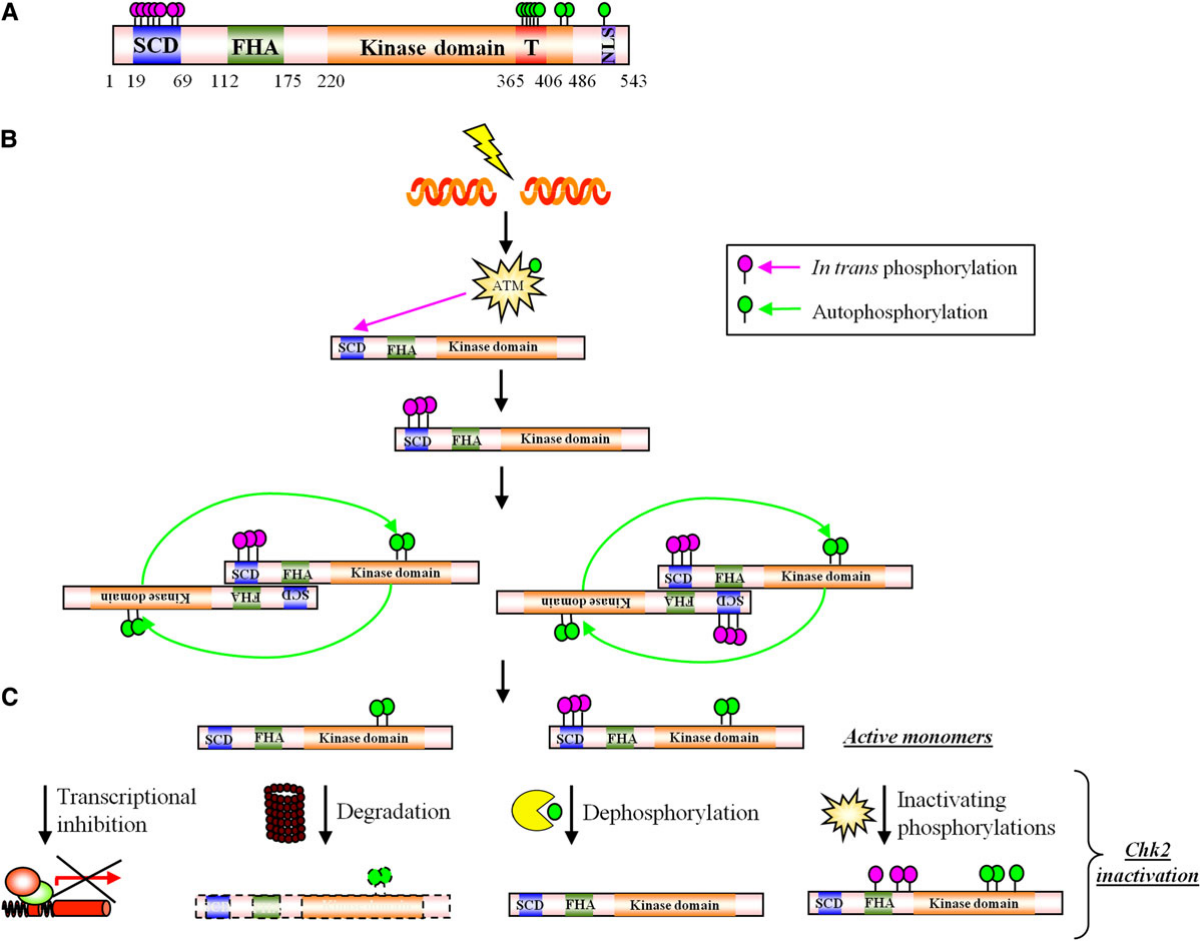
CHK2 activation and inactivation. (**A**) CHK2 protein primary structure. (**B**) After DNA damage, CHK2 monomers are phosphorylated in the SQ/TQ rich region, dimerize, and become active upon autophosphorylation. Successively they dissociate into active monomers. (**C**) CHK2 inactivation is achieved by degradation, dephosphorylation, and inactivating phosphorylations.

CHK2 was discovered in 1998 as the mammalian homolog of *Saccharomyces cerevisiae* Rad53 and *Schizosaccharomyces pombe* Cds1 kinases that are active in the yeast DDR (Matsuoka et al., 1998). The protein is conserved in mouse, rat, zebrafish, *Xenopus laevis*, *Drosophila melanogaster*, and *Caenorhabditis elegans*. In humans, it is a single 65 kDa polypeptide of 543 residues with three distinct functional domains (Figure 2A). At the N-terminus, there is a region rich in serine-glutamine and threonine-glutamine pairs, called SQ/TQ cluster domain (SCD); these SQ/TQ motifs are sites of phosphorylation by PI3K family kinases including ATM and ATR (Buscemi et al., 2006; Matsuoka et al., 2007). Between residues 112 and 175, a forkhead-associated (FHA) domain is responsible for the interactions with phosphorylated proteins, including the phosphorylated SCD of another CHK2 molecule (Li et al., 2002). Both SCD and FHA domains are common components of DDR proteins. In the C-terminal half of CHK2, a canonical kinase domain spans residues 220–486. Like many protein kinases, CHK2's catalytic function is activated by the phosphorylation of a polypeptide region (named activation loop or T-loop; residues 366–406) that lies inside the kinase domain but outside the active-site cleft. The T-loop contains several residues that undergo autophosphorylation for efficient kinase activity (Guo et al., 2010). Finally, between residues 515 and 522, there is a nuclear localization signal that targets newly synthesized CHK2 to this subcellular compartment (Zannini et al., 2003).

### Activation of CHK2

During normal growth, CHK2 is present in the nucleus in an inactive monomeric form (Ahn et al., 2000). After DNA damage, CHK2 is phosphorylated by ATM on the priming site T68 and on other residues in the SCD (Figure 2B). These phosphorylations lead to a conformational change which induces CHK2 dimerization through binding of the phosphorylated SCD of one monomer with the FHA domain of another (Ahn et al., 2002; Xu et al., 2002). Dimerization promotes CHK2 autophosphorylation of the kinase domain at residues S260 and T432, the T-loop residues T383 and T387, and S516 (Lee and Chung, 2001; Schwarz et al., 2003; Wu and Chen, 2003), triggering an additional conformational change and dissociation of the dimers into fully active monomers. Although phosphorylation of the SCD is the initial, essential step of CHK2 activation, this domain is rapidly dephosphorylated, perhaps because after dimer dissociation it is exposed to phosphatases (Ahn et al., 2002). Therefore, SCD phosphorylation is detectable only at early time points after damage.

Structural information about CHK2, from crystallography and mass spectrometry, has permitted the discovery of new phosphorylation (King et al., 2006; Guo et al., 2010) and ubiquitinylation events (Lovly et al., 2008) involved in the activation of CHK2. A strict, spatiotemporally regulated sequence of phosphorylations within the activating T-loop was shown to control CHK2 kinase activity and modulate its recognition of phosphorylation targets and its localization on chromatin (Guo et al., 2010). This study showed that CHK2 is transiently retained on broken chromatin, suggesting that it also participates in the repair of lesions. Another study reported that CHK2 autophosphorylates on Ser379, an event that facilitates CHK2 ubiquitinylation by an E3 ligase complex containing Cullin 1 (Lovly et al., 2008).

CHK2 may also be activated by DNA-dependent protein kinase (DNA-PKcs; Li and Stern, 2005), another member of the PI3K family. DNA-PKcs was shown to phosphorylate exogenous CHK2 in undamaged BJ-hTERT immortalized human fibroblast cells (Buscemi et al., 2009). After DNA damage, it phosphorylates a subfraction of CHK2 molecules bound to chromatin or centrosomes (Shang et al., 2010), preventing mitotic catastrophe. These findings suggest that DNA-PKcs participates in the activation of CHK2, at least when damage occurs during mitosis*.* In addition, upon DNA damage, Polo-like kinase-3 (PLK3), which phosphorylates CHK2 at S62 (in the SCD) and at S73 (Bahassi el et al., 2006), and the DNA mismatch repair protein MSH2, which interacts with CHK2 at sites of damage (Adamson et al., 2005), facilitate ATM-mediated phosphorylation of T68 and promote CHK2 activation. CHK2 autophosphorylation seems to also be regulated by PML protein (Yang et al., 2006), a tumor suppressor implicated in acute promyelocytic leukemia and a main component of PML-nuclear bodies (PML-NBs), which are nuclear matrix-associated structures. PML-NBs appear to be storage sites for inactive CHK2, which leaves the structures when activated (Yang et al., 2002). Nonetheless, a fraction of active CHK2 is retained in PML-NBs, where it phosphorylates PML protein itself or associates with p53 to regulate PML-NB number and PML-induced apoptosis (Yang et al., 2002; Zannini et al., 2009).

### Inactivation of CHK2

While much is known about CHK2 activation, many aspects of its inactivation remain to be elucidated. In the absence of DNA damage, CHK2 is maintained in an inactive state by serine/threonine protein phosphatase 2A (PP2A; Freeman et al., 2010) protein phosphatase 1D (WIP1; Fujimoto et al., 2006) and serine/threonine protein phosphatase 1 (PP1; Carlessi et al., 2010). After the DDR has run its course, CHK2 must be deactivated but it is not known to what extent this happens by degradation, dephosphorylation or phosphorylation at inactivating sites (Figure 2C).

Evidence that CHK2 is degraded at the end of the DDR comes from work in the cervical cancer cell line HeLa, where CHK2 levels dropped at just 1 h after irradiation (Schwarz et al., 2003) and in the A2780 ovarian cancer cell line where it was degraded in response to cisplatin treatment (Zhang et al., 2005). Moreover, a recent study showed that, in mice, CHK2 phosphorylated on S460 (corresponding to S456 in humans) is ubiquitinylated by p53-induced RING-H2 protein (PIRH2) and degraded by proteasomes (Bohgaki et al., 2013). In contrast, CHK2 accumulated in the non-small cell lung carcinoma cell line NCI-H460 after exposure to IR (Zhang et al., 2006) and its stability after DNA damage increased by phosphorylation on S456 in HCT-15 colon cancer cells (Kass et al., 2007). These conflicting results indicate that CHK2 protein levels are differently regulated depending on the cell line and on the type of the genotoxic agent.

CHK2 may also be deactivated by dephosphorylation by the phosphatases that normally maintain this protein in an inactive state in the absence of DNA damage, such as PP2A (Freeman et al., 2010) and WIP1 (Fujimoto et al., 2006; Oliva-Trastoy et al., 2007). A third way in which CHK2 is deactivated involves phosphorylation of the FHA domain by Polo-like kinase-1 (PLK1; van Vugt et al., 2010) that reduces its ability to bind phosphorylated proteins, including other CHK2 molecules.

### CHK2 substrates

When activated, CHK2 phosphorylates nuclear proteins involved in many aspects of the DDR. So far, 24 proteins have been described as CHK2 substrates in human cells (Table 1) and many of them fall into one of four functional groups involved in DNA repair, cell cycle regulation, p53 signaling, and apoptosis. CHK2 phosphorylates these substrates on one or more serine or threonine residues; however, for a few substrates the phosphorylated residues have not been identified. For many of these proteins, phosphorylation occurs at an RXXS or RXXT motif (Seo et al., 2003), the same sequence phosphorylated by calcium/calmodulin-dependent protein kinase II, cAMP-dependent protein kinase A, RAC-α serine/threonine protein kinase and others. Since not all CHK2 substrates contain this sequence, and because *in vitro* CHK2 phosphorylated related motifs containing basic residues upstream of a serine or threonine (Mendoza et al., 2013), further research is needed to understand the biochemical features of CHK2 substrates.

**Table 1:** Proteins phosphorylated by CHK2 in response to DNA damage, by functional category.

	Chk2 substrate	Phosphorylation sites	RXXS or RSST motif	Biological function	ATM target
DNA repair					
	BRCA1	S988	No	HDR and NHEJ	Yes
	BRCA2	T3387	Yes	HDR	Yes
	XRCC1	T248	No	BER	NA
	FOX-M1	S361	Yes	BER	NA
	KAP-1	S473	Yes	Chromatin reorganization	Yes
Cell cycle regulation					
	CDC25A	S123	Yes	G1/S checkpoint	NA
	LATS2	S408	Yes	G1/S checkpoint	NA
	Rb	S612	No	G1/S checkpoint, apoptosis repr.	NA
	CDC25C	S216	Yes	G2/M checkpoint	NA
	TTK/hMPS1	T288, S281	No, No	G2/M checkpoint	NA
p53 signaling					
	p53	T18, S20	No, No	Apoptosis	Yes
	HDMX	S367, S342	Yes, No	p53 accumulation	Yes
	CABIN1	NA	NA	p53 activation on chromatin	Yes
	pVHL	S111	Yes	p53 activation	NA
	STRAP	S221	Yes	G2/M checkpoint	Yes
	CHE-1	S141, S474, S508	Yes, Yes, Yes	G2/M checkpoint	Yes
Apoptosis					
	PML	S117	Yes	Apoptosis	NA
	E2F1	S364	Yes	Apoptosis	Yes
	HuR	S88, S100, T188	Yes, Yes, No	Apoptosis or prosurvival	NA
Other or unknown role					
	PP2A	NA	NA	CHK2 inactivation?	NA
	TRF2	S20	Yes	NA	Yes
	BLM	NA	NA	NA	Yes
	TAU	S262	No	NA	NA
	CDK11	S737	Yes	Pre-mRNA splicing	NA

Some CHK2 substrates contain the RXXS or RSST phosphorylation motif and some are also phosphorylated by serine/threonine protein kinase ATM.

NA, information not currently available.

Several proteins phosphorylated by CHK2 are also substrates of ATM, including BRCA1, BRCA2, KAP-1, and p53 (Banin et al., 1998; Gatei et al., 2000; Wang et al., 2004; White et al., 2006; Matsuoka et al., 2007) suggesting that CHK2 reinforces or redirects ATM function. Despite the identification of >20 CHK2 substrates so far, a large-scale proteomics analysis of cellular proteins phosphorylated by this kinase, as has been done for ATM and ATR (Matsuoka et al., 2007), has not yet been reported. Such a study would help clarify roles of CHK2 in the DDR and in normal cell physiology.

## Multiple roles of CHK2 in nuclear DNA damage repair

### Repair of DSBs and base modifications

Eukaryotic cells possess two systems to repair and rejoin broken DNA ends (Ciccia and Elledge, 2010): non-homologous end joining (NHEJ) and homology directed repair (HDR). NHEJ is involved in the repair of DSBs caused by endogenous and exogenous genotoxic agents and has an important role in the repair of programmed DSBs in normal mammalian cells, like during V(D)J and class-switch recombination (Lieber, 2010). HDR is more accurate than NHEJ, but requires the presence of an undamaged homologous template. Since sister chromatids are preferred to homologs, probably because of proximity, HDR occurs preferentially during S and G2 phases.

The relative extent to which DSBs are repaired by these two systems depends on the species and cell type (Iyama and Wilson, 2013). In simple eukaryotes with compact genomes, HDR is preferred. In mammals, where intergenic spacers and repetitive regions are abundant, NHEJ can be more efficient and for this reason, it is largely used in human cells (Iyama and Wilson, 2013) whereas HDR is confined to a backup function dedicated to lesions difficult to be repaired.

**Figure 3:**
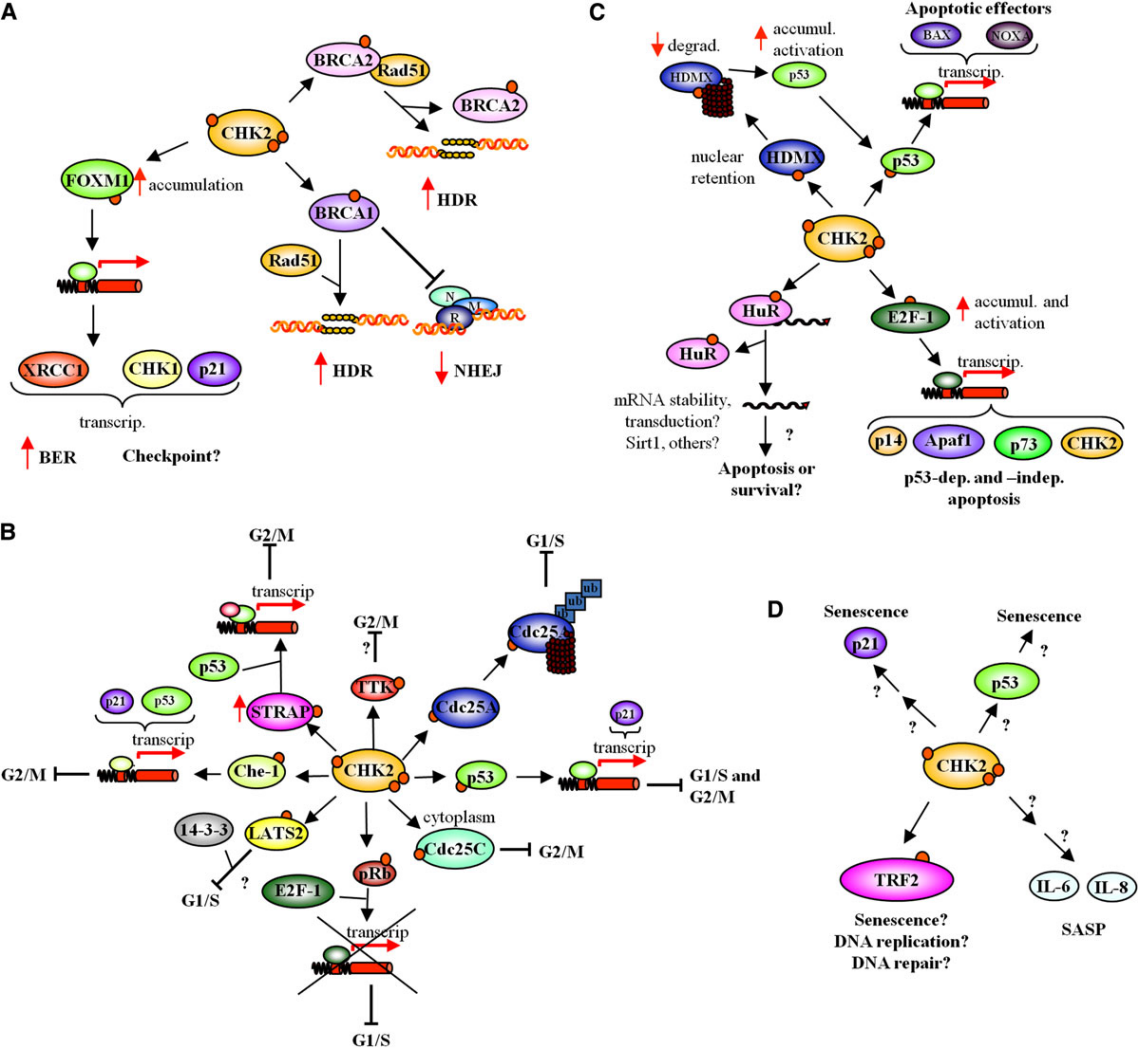
Multiple roles of CHK2 in nuclear DNA damage response. (**A**) CHK2 in the repair of damaged DNA. CHK2 phosphorylates BRCA1 and BRCA2 to regulate HDR and NHEJ, and phosphorylates FoxM1 to promote FoxM1 accumulation and subsequently BER. N,M,R is the Nbs1/Mre11/Rad50 complex. (**B**) CHK2 in cell cycle checkpoint activation upon DNA damage. CHK2 phosphorylates p53, Cdc25A, Lats2, and Rb to promote G1/S arrest and phosphorylates p53, Cdc25C, Che-1, Strap, and TTK to induce G2/M checkpoint activation. (**C**) CHK2 in apoptosis. Upon DNA damage, CHK2 phosphorylates p53 and MdmX to promote p53 accumulation and p53-dependent apoptosis. CHK2 targets E2F1 to induce both p53-dependent and independent apoptosis, and phosphorylates HuR to modulate apoptosis and survival. (**D**) CHK2 in senescence. CHK2 regulates senescence by targeting TRF2 and possibly acting on p53, p21, and IL-6 and IL-8.

CHK2 directly participates in the early steps of DSB repair by phosphorylating the two breast cancer susceptibility proteins, BRCA1 (Lee et al., 2000) and BRCA2 (Bahassi et al., 2008), with the final outcome of promoting HDR over NHEJ (Figure 3A). On one hand, after DNA damage, CHK2 phosphorylation of BRCA1 facilitates recruitment of the recombinase Rad51 to the lesion and repression of the NHEJ functions of the exonuclease Mre11 (Zhang et al., 2004). Rad51 then promotes DNA strand invasion and the exchange steps (Ciccia and Elledge, 2010), which are the main events of HDR. On the other hand, CHK2 phosphorylation of BRCA2 leads to disruption of the Rad51-BRCA2 complex, also allowing Rad51 to bind lesioned sites (Bahassi et al., 2008). Because Rad51 is a key component of the HDR, these phosphorylation events favor this repair pathway.

CHK2 has also been implicated in base excision repair (BER), a mechanism by which damaged nucleobases are recovered. Indeed CHK2 phosphorylates, and activates, the transcription factor forkhead box protein M1 (FoxM1, Figure 3A) which in turn induces the transcription of the base excision repair factor XRCC1 (Tan et al., 2007).

These findings indicate that CHK2, like other proteins (e.g. PARP1, ATM, ATR, p53, BLM, and BRCA2), is involved in different repair processes and underline the strong connection among repair pathways, which cooperate in the restoration of DNA integrity.

### DSB repair and heterochromatin relaxation

The successful rejoining of DNA ends requires that DDR proteins can access the lesion within the complex chromatin ‘landscape’. DSBs occurring nearby or within heterochromatin are difficult to repair because the chromatin is more compact (Goodarzi and Jeggo, 2012). Therefore, DDR proteins modify histones and remodel the nucleosome to facilitate lesion processing. In this context, CHK2 ‘collaborates’ with ATM by phosphorylating a common substrate, the transcriptional repressor KRAB-associated protein 1 (KAP-1). To relax chromatin ATM phosphorylates KAP-1 at Ser824, disrupting the complex between KAP-1 and the nucleosome remodeler protein (CHD3) and allowing CHD3 to be removed or to dissociate from heterochromatin (Goodarzi et al., 2011). Then, CHK2 phosphorylates KAP-1 on Ser473, which compromises the KAP-1/HP1-β interaction on chromatin, leading to HP1-β mobilization (Bolderson et al., 2012; Hu et al., 2012). Since HP1-β is a heterochromatin packaging factor, the release of this protein from chromatin relaxes the DNA structure, further favoring the access of repair factors to lesion sites.

### Cell cycle arrest at checkpoints

Cell cycle checkpoints coordinate cell cycle progression with repair pathways. By transiently arresting or delaying the cell cycle, they provide the necessary time for the repair of a lesion before the critical phases of DNA replication and mitosis, when the genome is segregated.

In the presence of DSBs, CHK2 arrests the cell cycle at G1/S and G2/M by several mechanisms (Figure 3B). About G1/S arrest, one mechanism involves the phosphorylation and subsequent degradation of Cdc25A phosphatase by the proteasome; this event prevents the dephosphorylation and activation of cyclin-dependent kinase 2 (Cdk2), needed for G1/S and S phase progression (Falck et al., 2001). Another mechanism involves the phosphorylation of p53 by ATM and CHK2, which stabilizes and activates p53 (Chehab et al., 2000; Hirao et al., 2000)*.* Activated p53 upregulates the expression of p21, an inhibitor of cyclin-dependent kinases, leading to G1/S transition arrest (Chehab et al., 2000). However, because this pathway requires transcription of p21 gene, it is assumed to sustain, more than start, G1/S arrest (Bartek and Lukas, 2001).

The molecular mechanisms of CHK2-dependent G2/M arrest are similar to those of G1/S arrest (Figure 3B). In this case, CHK2 phosphorylates Cdc25C (Matsuoka et al., 1998), which results in the translocation of this phosphatase to the cytoplasm, through an interaction with 14-3-3 proteins (Dalal et al., 1999). In the cytoplasm, Cdc25C can no longer dephosphorylate and activate the cyclinB1/Cdk1 complex, necessary for the G2/M transition (Takizawa and Morgan, 2000). CHK2 also phosphorylates p53 to promote p21 accumulation and sustain G2/M arrest (Hirao et al., 2000). However, the role of CHK2 in p53-mediated p21 accumulation is disputed, since there are contrasting observations from CHK2 knockout mice (Jack et al., 2002; Takai et al., 2002) as explained below.

CHK2 also seems to impose cell cycle arrest through other pathways (Figure 3B) whose relevance to the DDR is unknown. For example, CHK2 phosphorylates pRb which enhances the formation of the transcriptionally inactive pRb/E2F-1 complex causing G1/S arrest and apoptosis repression (Inoue et al., 2007). Furthermore, CHK2 phosphorylates the serine/threonine protein kinase LATS2 probably inducing G1/S arrest (Okada et al., 2011). Three other additional mechanisms leading to G2/M arrest have been proposed. In the first, CHK2 and ATM phosphorylate the apoptosis antagonizing transcription factor Che-1, which results in its stabilization and recruitment to the promoters of p21 and p53 genes after damage, leading to their transcription and to the sustainment of G2/M arrest (Bruno et al., 2006). In the second, ATM phosphorylates the serine/threonine kinase receptor-associated protein STRAP, a p53 cofactor, promoting its nuclear localization, whereas STRAP phosphorylation by CHK2 augments its stability, inducing a p53-dependent G2/M arrest (Adams et al., 2008). Finally, CHK2 phosphorylates and stabilizes the dual specificity protein kinase TTK/hMps1, promoting G2/M arrest by an unknown mechanism (Yeh et al., 2009).

In eukaryotic cells an S-phase checkpoint, dedicated to replicative fork surveillance, exists. However, CHK2 has not been shown to be associated with replication fork arrest, which is mainly under the supervision of ATR-CHK1 pathway (Paulsen and Cimprich, 2007).

### Triggering apoptosis in cases of irreparable DNA damage

Cells with irreparable damage activate apoptotic pathways, to avoid propagation of a modified, potentially harmful genome. The induction of apoptosis proceeds through at least two main pathways (extrinsic and intrinsic), each of which can be regulated at multiple levels. A common regulator of both these apoptotic pathways is p53, a transcription factor and tumor suppressor protein that, in response to DNA damage, induces the expression of genes involved in checkpoint activation or apoptosis (Gomez-Lazaro et al., 2004). In unstressed cells, p53 has low activity and a short half-life because it is complexed with two proteins, the E3 ubiquitin protein ligase (MDM2) and the Mdm2-like p53-binding protein (HDMX), which cause p53 to be ubiquitinated and degraded by proteasomes; low p53 levels and activity allow normal growth (Gomez-Lazaro et al., 2004). After DNA damage, p53 is phosphorylated by ATM, an event that displaces MDM2 and allows p53 to accumulate in the nucleus where it can perform its function as transcription factor (Cheng and Chen, 2010). This p53 stabilization is also due to the degradation of HDMX, which is induced by phosphorylation by both ATM and CHK2 (Chen et al., 2005b; LeBron et al., 2006; Pereg et al., 2006). Indeed, HDMX normally shuttles between the nucleus and the cytoplasm, but in the presence of DSBs, nuclear HDMX is phosphorylated by ATM and CHK2 and retained there through binding to 14-3-3 proteins (Chen et al., 2005b). This event is an essential step toward HDMX degradation, p53 activation and apoptosis induction (Figure 3C).

In cells exposed to IR, p53 and histone H1.2 were also found to translocate to the mitochondria where they directly induced apoptosis. This event was markedly reduced in CHK2-deficient cells due to defective p53 stabilization (Chen et al., 2005a).

CHK2 can also induce apoptosis through the phosphorylation and stabilization of the transcription factor E2F-1, which activates the transcription of proapoptotic genes (Figure 3C; Stevens et al., 2003). This pathway is reinforced by E2F-1-activated CHK2 transcription (Powers et al., 2004). Furthermore, CHK2 can induce apoptosis by phosphorylating Hu-antigen R (HuR), a protein involved in binding and stabilizing mRNAs. In human WI-38 fibroblast cells subjected to oxidative stress, CHK2 phosphorylated HuR, thereby triggering the dissociation of HuR from the mRNA encoding NAD^+^ dependent protein deacetylase sirtuin 1 (SIRT1), which is involved in the promotion of cell survival upon stress (Abdelmohsen et al., 2007). This event facilitated the degradation of SIRT1 mRNA, reduced the abundance of SIRT1 protein, and promoted apoptosis. However, in human colorectal carcinoma cells subjected to radiation, CHK2 induced the dissociation of virtually all HuR-mRNA complexes, including those encoding apoptotic and mitogenic proteins (Masuda et al., 2011). In this case, HuR phosphorylation by CHK2 helped implement appropriate gene expression patterns that enhanced the survival of cells with damaged DNA.

Finally, it has recently been uncovered an unexpected role for CHK2 in the promotion of anoikis (Yoo et al., 2012), a form of cellular self-killing that occurs specifically when cells are detached from the extracellular matrix.

## CHK2–p53 interplay

As described in the previous sections, CHK2 seems to directly and indirectly regulate p53 activity. However, conflicting data exist about the direct relationship between CHK2 and p53. A study published in 2000 reported that, upon DNA damage, CHK2 phosphorylates p53 on Ser20 (Chehab et al., 2000). Subsequent studies disputed this finding (Ahn et al., 2003; Jallepalli et al., 2003). It remains unclear today whether CHK2 phosphorylates p53 to induce pro-survival or pro-apoptotic pathways. Furthermore it is elusive whether CHK2 phosphorylates p53 after this protein is stabilized by ATM in response to DSBs or if it phosphorylates a latent p53 fraction that existed before DNA damage (Jack et al., 2002, 2004).

CHK2 can also activate p53 via the phosphorylation of other proteins, such as Che-1, STRAP, HDMX, as described above, and calcineurin binding protein 1 (CABIN1). CABIN1, in unstressed conditions, keeps p53 in an inactive state on chromatin. In human HCT116 colon cancer cells, the induction of DNA damage caused CHK2 (and ATM) to phosphorylate CABIN1, promoting its degradation and freeing p53 from inhibition (Choi et al., 2013). Additionally, in HCT116 cells in response to DSBs, CHK2 phosphorylated the von Hippel–Lindau tumor suppressor (VHL, the protein mutated in von Hippel–Lindau disease, a hereditary cancer syndrome; Roe et al., 2011). Phosphorylated VHL then associated with an acetylase (either p300 or Tip60) and the complex bound p53 at chromatin, leading to p53 transactivation.

## CHK2 and senescence

Normal diploid cells have a limited ability to proliferate in cell culture: after a period of growth they cease to divide and enter a state of cellular senescence. This phenomenon has, for a long time, been considered the result of an exhaustion of the proliferative lifespan (replicative senescence; Ohtani et al., 2009). More recently, senescence has been described as a barrier to the replication of cells chronically exposed to damaging insults (premature senescence) or of cells with activated oncogenes that stimulate replication and generate DNA lesions (oncogene-induced senescence, OIS; Gorgoulis and Halazonetis, 2010). Both replicative senescence and OIS can be triggered by telomere shortening. Telomeres, chromosomal ends that are structurally similar to DNA breaks, are under the control of the DDR. Normal telomeres are protected by a protein complex named shelterin that binds the repeated telomeric DNA sequence (de Lange, 2005). Shelterin proteins (in humans, TRF1, TRF2, TPP1, POT1, TIN2, and RAP1) create a three-dimensional structure called telomere loop that hides chromosomal ends from exonucleases, damage sensors, and repair proteins, thereby preventing chromosome fusions. Moreover, shelterin proteins inhibit the main DDR kinases: TRF2 is a repressor of ATM and CHK2 (Karlseder et al., 2004; Buscemi et al., 2009), masking their activation domains, while POT1 has the same effect on ATR (Denchi and de Lange, 2007). Telomere stress or shortening partially uncovers telomeres and creates an opportunity for ATM and CHK2 to function (Di Micco et al., 2006), finally leading to a permanent arrest of the cell cycle and to the acquisition of senescence features, like cellular flattening and vacuolization (Kuilman et al., 2010). The substrates phosphorylated by CHK2 to start the senescence program are currently unknown, but cells in which CHK2 was overexpressed had features of senescence that seemed to be p53 independent and p21 dependent (Aliouat-Denis et al., 2005).

In the presence of DNA damage, ATM and CHK2 phosphorylate TRF2, reducing its affinity for telomeres (Figure 3D; Tanaka et al., 2005; Buscemi et al., 2009), but whereas ATM promotes TRF2 relocalization from telomeres to the DNA lesion, probably to enhance the protection and repair of the DSB, the functional significance of CHK2 phosphorylation of TRF2 is unclear.

CHK2 has also been described to be an essential player in a process named senescence-associated secretory phenotype (SASP), in which senescent cells express and secrete numerous proteins that alter the local tissue environment (Figure 3D; Coppe et al., 2010). The expression of several SASP proteins, particularly the inflammatory cytokines IL-6 and IL-8, is regulated by a pathway involving CHK2, ATM, and the Nijmegen breakage syndrome protein NBS1, another DDR component (Rodier et al., 2009). Senescence is also associated with persistent nuclear foci containing DDR proteins, called DNA segments with chromatin alterations reinforcing senescence (DNA-SCARS). These structures, in which active CHK2, PML, and p53 coexist, regulate senescence-associated growth arrest (Rodier et al., 2011), IL-6 secretion, and sustainment of senescence after DNA damage.

## CHK2 and the mitotic catastrophe

When DNA damage occurs in G2 phase, CHK2 normally arrests the cell cycle at the G2/M boundary (Matsuoka et al., 1998). When CHK2 was repressed by expression of an inactive dominant-negative CHK2 mutant or by exposure to specific chemical inhibitors, HCT116 colon cancer cells with DNA lesions entered mitosis and, in metaphase, underwent apoptosis (Castedo et al., 2004), a phenomenon called mitotic catastrophe (Castedo and Kroemer, 2004). In HeLa cells, upon DNA damage, Ku70/80 drives DNA-PKcs to phosphorylate SCD of CHK2 on centrosomes, kinetochores, and midbodies, stabilizing centrosomes and spindle formation in an unknown way and preventing mitotic catastrophe (Shang et al., 2010). These observations underline a role for CHK2 in monitoring mitotic structures, an activity further confirmed by studies in the absence of exogenous damage.

## DDR activities: specificity, flexibility, redundancy

**Figure 4:**
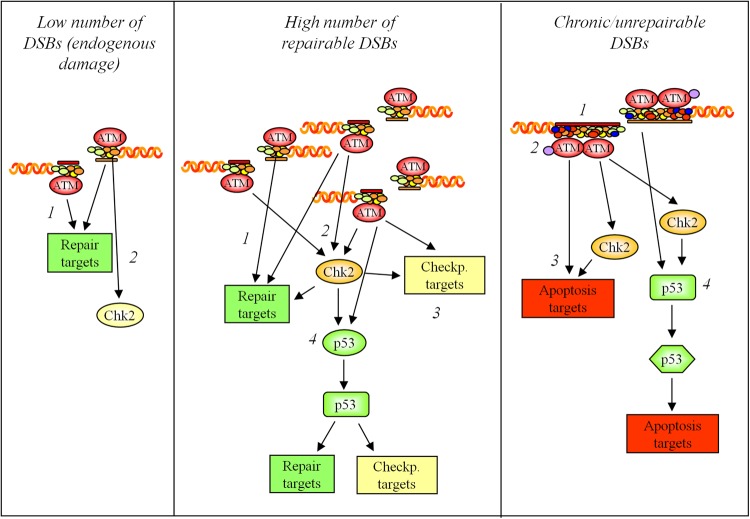
Molecular events that allow CHK2 to sense and respond to different levels of DNA damage.

From the findings summarized above, it is clear that the DDR is complex at the molecular level. This complexity reflects not only its importance for survival but also the need for a highly specific, modulated response. Indeed, cells that experience a few DSBs can repair the damage with no or only a modest cell cycle delay (Ciccia and Elledge, 2010; Figure 4). However, after exposure to genotoxic agent, the repair of a large amount of damage requires cell cycle arrest or even senescence or apoptosis after the first attempts to repair DNA. Various DDR pathways are activated according to the extent and the characteristics of the lesions. Therefore, the apical pathways, like the ATM-CHK2 axis, can be specific but flexible to drive repair, cell cycle arrest, apoptosis, or senescence in response to the burden of DSBs. Such flexibility is due to changes in kinase-substrate affinity that depends on the availability of active kinase, the presence of specific recruiting or coactivating factors, and the availability, accessibility and status of the substrates.

The importance of the DDR for the survival of an organism has evolutionarily added another level of complexity that is the high redundancy and cooperation within the DDR pathways. For these reasons, although the DDR and involvement of CHK2 have been studied for years, many aspects of this physiological complexity remain elusive. For example, the definition of lesion and genotoxic dose specificity (Buscemi et al., 2004) in relation to the final biological outcomes are essential for identifying proteins that can be targets of therapy for DDR-dependent syndromes and cancer, but remain up to now undefined. The complexity of the DDR is further complicated by the many variables present in any experimental approach. It is difficult to experimentally assess the lesion specificity of DDR components using *in vitro* cultured cells since genotoxic treatments generally induce primary and secondary damages depending on the cell cycle phase and extent of exposure. Other sources of complexity are variations among cell types in DNA repair mechanisms, predisposition to cell cycle arrest or death, genetic background, epigenetic status, cell cycle phase, and cell age. Finally, lesions produced by γ-radiation or radiomimetic drugs are not necessarily the same, even though these agents are used indifferently. Thus, the identification of a threshold dose for the activation of prosurvival, senescence or cell death programs is even more complex.

## Roles of CHK2 in normal cellular physiology

### CHK2 monitoring of mitosis and meiosis

**Figure 5:**
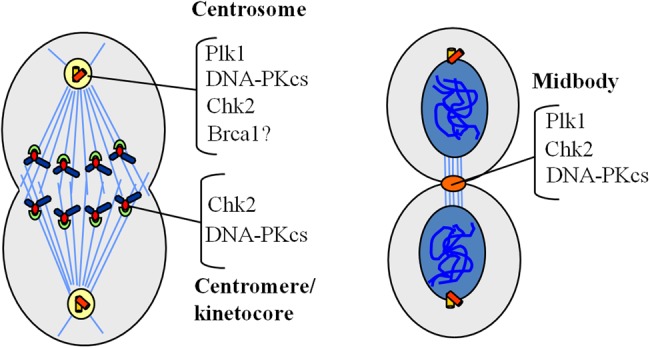
Functional CHK2 interactors on specialized structures during mitotic phases.

A DNA lesion occurring in S or M phase can be exacerbated or fixed; therefore these transitions are strictly monitored. Whereas in the absence of DNA stress the ATR-CHK1 pathway guards S phase progression (Maya-Mendoza et al., 2007), CHK2 monitors M phase (Stolz and Bastians, 2010). CHK2 depletion or inactivation in colon cancer cells caused abnormal spindle assembly, mitotic delay, and chromosome instability, but allowed cell survival and growth (Stolz et al., 2010). However we do not know whether CHK2 acts on these events in the absence of DNA stress or whether the endogenous damage of highly proliferating cells induces CHK2 activity. BRCA1 is a direct target of CHK2 in the mitotic pathway and, coherently, a fraction of CHK2 and BRCA1 is localized on the centrosomes (Figure 5; Zhang et al., 2007). A small amount of phosphorylated CHK2 also colocalizes with PLK1 on the centrosomes and midbody (Tsvetkov et al., 2003) in the absence of damage, with an unknown function, but further reinforcing the role of CHK2 in mitosis control. It is also unclear whether this pathway and that regulated by DNA-PKcs in response to DNA damage (see ‘CHK2 and the mitotic catastrophe’ section) are linked or completely independent.

Other CHK2 activities independent of exogenous damaging sources have been described to occur during meiosis in experimental models. In *C. elegans,* CHK2 is essential for meiosis since it participates in chiasma formation and in crossover events (Higashitani et al., 2000). In *D. melanogaster* a role for CHK2 at the meiotic checkpoint is controversial (Abdu et al., 2002; Masrouha et al., 2003). In the same organism, the absence of CHK2 leads to the accumulation of abnormal nuclei in the syncytial embryo, resulting probably from defects in arresting or eliminating cells with endogenous DNA damage (Pushpavalli et al., 2013). In mice, CHK2 is activated by ATM in female germ cells during early meiosis (Miles et al., 2010) and regulates cell cycle progression and spindle assembly during oocyte maturation and early embryo development (Dai et al., 2014). Moreover, CHK2 induces the removal of oocytes with unrepaired meiotic or induced DSBs by activating p53 and p63. Thus, CHK2 ablation can reverse female mice infertility caused by meiotic recombination defects or irradiation (Bolcun-Filas et al., 2014).

### CHK2 in stem cells

The characterization of the DDR in stem cells is important not only for basic knowledge but also for technical and therapeutic reasons. However, current knowledge about the DDR in stem cells is limited. It is known that mutations in DDR proteins lower the possibility of success in the stabilization of induced pluripotent stem (iPS) cells, and also that p53 is transiently inactivated during the process of iPS cell generation (Hong et al., 2009; Marion et al., 2009).

CHK2 and other DDR proteins were found to be highly expressed in both human embryonic stem (ES) and iPS cells (Momcilovic et al., 2010). This is coherent with the high repair efficiency detected in these cells and obtained essentially by HDR. Overexpression of DDR proteins could also explain why cancer stem cells are resistant to radiation, an event that can be reversed by CHK1 and CHK2 inhibition (Bao et al., 2006). Indeed, in ES cells, the G1 checkpoint is absent; accordingly, in these cells, CHK2 was unable to induce Cdc25A degradation (Hong et al., 2007). On the other hand, apoptosis occurs more frequently in ES than in somatic differentiated cells, perhaps as a safety mechanism to prevent the replication of a mutated genome (Hong and Stambrook, 2004).

### CHK2 and viral infection

**Figure 6:**
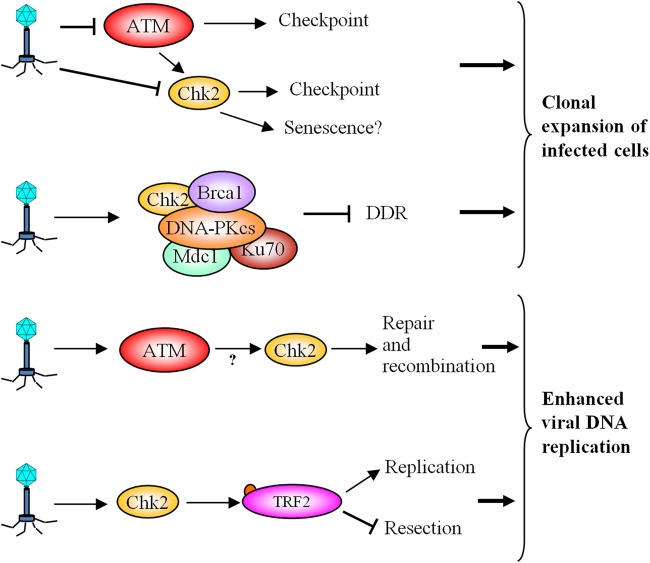
CHK2 in viral infection. Viruses can alter cell cycle control and DNA replication, with important consequences on the DDR.

The ability of viruses to manipulate the environment for their own needs is counteracted by the host's defense system. Viruses can alter cell cycle control and DNA replication, with a consequent effect on DDR (Turnell and Grand, 2012). Each virus has different effects and the same virus can activate or inhibit the DDR at different stages of infection (Figure 6). For the simian virus 40, herpes simplex virus, cytomegalovirus, and Epstein-Barr virus (EBV), viral DNA synthesis depends on the recruitment and activation of ATM and DNA repair pathways. In contrast, adenoviruses induce the degradation of DDR proteins like p53, BLM, and Mre11, leading to the repression of DDR and of apoptosis. G1 checkpoint inactivation is particularly important since viruses lack many of the proteins required for DNA replication, such as polymerases, which in hosts accumulate during S phase (Clark et al., 2000; Moody and Laimins, 2009, 2010).

During EBV infection, the nuclear antigen 3C (EBNA3C) directly interacts with CHK2, inhibiting G2/M arrest (Choudhuri et al., 2007). EBNA3C is essential for immortalization of primary B lymphocytes *in vitro*, a complex event that reflects the ability of many viruses to prevent senescence in host cells. As proposed by Reddel (2010), the repression of senescence by viruses, counteracted by the cellular production of SASP, suggests that senescence was an ancestral antiviral defense mechanism that prevented the infection of proximal cells. An interesting connection among telomeres, DDR and viral DNA replication has been described during latent EBV infection (Zhou et al., 2010). TRF2 is recruited to the EBV origin of replication (OriP) to favor DNA replication and perhaps to repress recombination or resection by host DDR. At the same time, CHK2 phosphorylates TRF2 during S phase, to dissociate TRF2 from OriP and stabilize episomal DNA by an undefined mechanism (Zhou et al., 2010).

Another example of a CHK2-virus connection involves the human T-cell leukemia virus, type I (HTLV-1). The viral Tax protein binds and sequesters DNA-PKcs, Ku70, MDC1, BRCA1, and CHK2, forming DNA damage-independent nuclear foci and competing with the normal DDR (Durkin et al., 2008; Belgnaoui et al., 2010). Consequently, cells do not sense damage and divide without restrictions, increasing the number of infected cells. On the other hand, repression of DNA repair pathways by HTLV-1 induces genomic instability in the host, supporting cellular transformation to T-cell leukemia.

### CHK2 and mitochondrial DNA damage

Damage to mitochondrial DNA (mtDNA) is generally considered marginal compared with nuclear DNA*.* In eukaryotic cells there are ∼80–700 mitochondria per cell, depending on the cell type, and each mitochondrion contains 2–10 copies of a small (16500 bp) heteroplasmic DNA (Tann et al., 2011). Therefore, the occurrence and transmission of mutations leading to respiratory chain defects and mitochondrial syndromes are rare and principally due to errors in mtDNA replication, more than damage (Park and Larsson, 2011). However, mtDNA is particularly vulnerable because it lacks protective histones and is fully coding due to the absence of introns. Moreover, it is in close proximity to the inner mitochondrial membrane, where reactive oxygen species and their derivatives are produced.

In budding yeast, Tel1 and Rad53, the homologs of ATM and CHK2, respectively, sense and are activated by mitochondrial reactive oxygen species (mtROS), in the absence of nuclear DNA damage (Schroeder et al., 2013). These events finally lead to chromatin remodeling at telomeric regions, by inactivation of the histone demethylase Rph1p, and extension of life span (Schroeder et al., 2013). In human cells, failure to repair mtDNA damage has been shown to initiate apoptosis (Koczor et al., 2009). However, further evidence supports the existence of a ‘mitochondrial checkpoint’ regulated by the nuclear DDR and particularly by CHK2 (Koczor et al., 2009). How the signal of mtDNA damage reaches CHK2 in the nucleus is unknown.

### CHK2 and the circadian clock

The circadian clock programs daily rhythms and regulates, at the cellular level, many metabolic systems. Both the cell cycle and the circadian clock are intracellular oscillatory systems (Hunt and Sassone-Corsi, 2007), probably evolved in a concerted manner: it is not accidental that most eukaryotic cells in culture undergo division with a periodicity of about 1 day. Therefore, there is also a linkage between the DDR and the circadian clock (Sancar et al., 2010). Specifically, period circadian protein 1 (PER1; Gery et al., 2006), period circadian protein 3 (PER3; Im et al., 2010), and TIMELESS (Yang et al., 2010), all components of the human circadian clock, seem important for CHK2 activation and interact physically with this kinase. It is relevant to note that PER1 expression reduces the growth of cancer cell lines and was downregulated in human tumors (Gery et al., 2006). Altogether these findings underline the importance of circadian regulation for cellular functions and suggest that disruption of circadian clock proteins could lead to the development of cancer.

In the bread mold *Neurospora crassa*, transcription of the CHK2 ortholog PRD4 has a day/night cycle that peaks in the morning (Pregueiro et al., 2006). In response to DNA damage and in presence of light, this protein phosphorylates the frequency clock protein (FRQ; Pregueiro et al., 2006) thus signaling the presence of DNA damage to the circadian clock and resetting the circadian rhythm. In principle, this phenomenon could enhance DNA protection from, for example, the mutagenic effects of ultraviolet light.

## Effects of experimental and pathological CHK2 impairment

ATM is not essential for life at the cellular level even though it is highly responsive to low levels of DNA damage and has many functions in the DDR (Shiloh and Ziv, 2013). Accordingly the absence of CHK2, which mediates only a subset of ATM activities, gives only mild and elusive phenotypes. Possible explanations are that ATM can start safeguarding programs independently of CHK2, or that CHK1 can partially compensate for the absence of CHK2.

Consequently, CHK2 deletion had no or only mild phenotypic effects on the majority of *in vitro* cultured normal human cell lines, exposed or not to physiological doses of damaging agents. However, analysis of different cell types has uncovered some defects due to the absence of CHK2. For example, thymocytes from CHK2 knockout mice were resistant to apoptosis in response to DNA damage (Takai et al., 2002), while in other cell lines this effect was not observed. Furthermore phenotypes associated with the absence of CHK2 seem more evident in cells where other DDR factors are impaired, for example in p53 defective cells.

In humans, CHK2 germline mutations have been detected with high incidence in a number of familial cancers, and rare somatic mutations have been reported in some tumors (Wu et al., 2001). In particular, two mutations leading to a truncated CHK2 protein with reduced or absent kinase activity, 1100delC and I157T, are low-penetrance cancer susceptibility mutations that increase the risk of developing breast, prostate, ovarian, colorectal, kidney, thyroid, and bladder cancers and leukemias (Wu et al., 2001). The increased risk is probably due to these mutations synergizing with a predisposed genetic background or with exposure to damaging factors like radiation. Overall, CHK2, more than a tumor suppressor, appears to function like a multi-organ tumor susceptibility gene (Cybulski et al., 2004).

In mice, no syndromes or cancer predisposition have been associated with the absence of CHK2, even though CHK2^−/−^ mice are more susceptible to skin tumors induced by carcinogenic agents and defects in the p53-dependent apoptotic pathway have been described in mouse embryonal fibroblasts (Hirao et al., 2002). In contrast, CHK1^+/−^CHK2^−/−^ and CHK1^+/−^CHK2^+/−^ mice had high levels of spontaneous DNA damage and failed to eliminate cells with lesions, prompting a progressive cancer-prone phenotype (Niida et al., 2010).

Differently from knock-out mice, knock-in mice expressing the CHK2*1100delC variant developed spontaneous lung and mammary tumors with shorter latency and higher frequency than wild type mice (Bahassi el et al., 2009). The majority of CHK2*1100delC-expressing mice with lung and mammary tumors were female, suggesting a gender bias in agreement with the hormonal responsiveness of these tissues. A possible influence of estrogen on CHK2 function is intriguing and can be ascribed to the activity of the estrogen receptor on the CHK2 target Cdc25A or to an interaction between the estrogen receptor and one of the proteins regulated by CHK2 or CHK2 itself. Another possibility is that the presence of high levels of estrogen metabolites increases the amount of DNA damage, through redox cycling processes, predisposing female mice with CHK2 mutations to cancer.

## CHK2 as a target for cancer therapy

As for other DDR components, CHK2 may be considered a good target for enhancing the therapeutic effect of DNA-damaging treatments in cancer. The scope of this type of treatment is to inactivate pro-survival DDR activities, such as DNA repair and cell cycle arrest, or activate senescence, apoptosis, or mitotic catastrophe programs preferentially in cancer cells. Although CHK2 was initially described as a regulator of DNA damage checkpoints, it was later found capable, if inhibited, to enhance the apoptotic activity of genotoxic agents. For this reason, small-molecule inhibitors of CHK2 have been evaluated in clinical trials in combination with other therapies (Bucher and Britten, 2008). However, the outcomes have been contrasting (Garrett and Collins, 2011). Indeed, the assessment of these molecules' anticancer efficacy can be confounded by the fact that CHK2 inhibitors are also often active on CHK1, which has a more defined prosurvival activity. To date, only CHK1-specific or dual-specificity CHK1/CHK2 inhibitors have entered clinical trials (Bucher and Britten, 2008; Matthews et al., 2013).

Conversely, it has been shown that CHK2 inhibition can provide protection from radiotherapy or chemotherapy (Jiang et al., 2009), probably as a consequence of its role in the induction of p53-dependent apoptosis. Thus, it is encouraging that CHK2 suppression could sensitize tumors with a p53-deficient background to DNA-damaging therapies. In fact, in this case, the contemporary absence of CHK2 and p53 leads to abrogation of both G1/S and G2/M checkpoints, hence sensitizing cells to genotoxic agents. In contrast, normal cells would be affected to a lesser extent since they retain normal cell cycle checkpoints and DNA repair pathways (Jiang et al., 2009).

It is also predictable that agents that perturb telomeric structures will be able to kill tumor cells that have longer telomeres than normal cells do. Indeed, drugs stabilizing G-quadruplexes at telomeres, which are four-stranded structures induced by the presence of consecutive guanines in the telomeric repeat sequence, trigger a potent ATM-CHK2 response although, unexpectedly, this leads to autophagy more than senescence or apoptosis (Zhou et al., 2009).

From this evidence, it is clear that, to kill cancer cells, the choice between CHK2 inhibition and activation depends on many variables such as the kind, magnitude and duration of exposure to the damaging agent, the genetic background of the cancer cells, and the specificity and efficacy of the CHK2 inhibitor. For these reasons, additional research is necessary before these molecules can be used to treat cancer.

## Conclusions and future directions

The role of human CHK2 in the DDR is not yet fully understood, and further studies are necessary to reconcile its different activities. Much has been disclosed about CHK2's function since its discovery, but much remains to be understood about its activation and, most of all, inactivation. In the next few years, new CHK2 substrates will probably be identified by proteomic approaches and wide screening analyses. Addressing the functional significance of every substrate in many cell types will be a challenging task that we need to conduct, keeping in mind the biological relevance and possible clinical applications.

We need to define those mechanisms and proteins that fine-tune the different biological outcomes of the DDR in relation to lesions, cellular types, and genetic background. Indeed, a more detailed knowledge of CHK2 activities in human cells in relation to damage type and extent could help define the possibility of treating specific tumors by CHK2 activation or inactivation, alone or in combination with other therapies. Particularly interesting is the possibility of targeting CHK2 in patients with known carcinogenic mutations in p53.

On the whole we need to define the variables and the conditions supporting the use of CHK2 inhibitors to treat cancer in a personalized manner. Moreover, a better knowledge of the response to virus infection or the relationship between DNA management and the circadian clock, could lead to the discovery of unexpected and intriguing aspects of cellular evolution.

## Funding

This work was financially supported by the Italian Ministry of Health (Project Code GR-2010-2315822) and by Italian Association for Cancer Research (AIRC, Project IG 10248).

**Conflict of interest:** none declared.
